# Examining Associations between Age, Binge Drinking, and Physical Activity Duration

**DOI:** 10.1093/geroni/igaf122.2737

**Published:** 2025-12-31

**Authors:** Abigail Nehrkorn-Bailey

**Affiliations:** University of Wisconsin-Green Bay, Green Bay, Wisconsin, United States

## Abstract

The existing literature suggests that engagement in one health risk behavior, such as alcohol consumption, is associated with other health risk behaviors, like physical inactivity. This association between health risk behaviors has been referred to as “clustering”. However, there are gaps in the current literature: (1) a significant portion of the binge drinking research has focused on the behavior in college-aged individuals and (2) Dodge et al. (2016) found a positive association between alcohol drinking and physical activity, which contradicts the idea of clustering. A two-way ANOVA was conducted, utilizing the July 2023 Behavioral Risk Factor Surveillance System data (CDC, 2023) to examine whether age (i.e., 18-39 years, 40-59 years, 60+ years) and binge drinking categories (i.e., 0 binge drinking episodes, 1-9 episodes, 10-29 episodes, and 30 or more episodes in the past month) were related to minutes of weekly physical activity engagement. Along with significant main effects, there was a statistically significant interaction between age and binge drinking on physical activity duration, F (6, 167478) = 3.07, p < .01. The interaction results are discussed in terms of the physical activity engagement differences found for different ages at each binge drinking level, highlighting the importance of assessing binge drinking and physical activity engagement across adulthood.

